# Exploring the dynamics of migration, armed conflict, urbanization, and anthropogenic change in Colombia

**DOI:** 10.1371/journal.pone.0242266

**Published:** 2020-11-24

**Authors:** Guibor Camargo, Andrés Miguel Sampayo, Andrés Peña Galindo, Francisco J. Escobedo, Fernando Carriazo, Alejandro Feged-Rivadeneira

**Affiliations:** 1 Facultad de Estudios Internacionales, Políticos y Urbanos, Universidad del Rosario, Bogotá, Colombia; 2 US Forest Service, Pacific Southwest Research Station, Riverside, CA, United States of America; Institute for Advanced Sustainability Studies, GERMANY

## Abstract

Anthropogenic change has been associated with population growth, land use change, and changing economies. However, internal migration patterns and armed conflicts are also key drivers of anthropogenic and demographic processes. To better understand the processes associated with this change, we explore the spatial relationship between forced migration due to armed conflict and changing socioeconomic factors in Colombia, a country which has a recent history of 7 million internal migrants. In addition, we use remote sensing, Google Earth Engine, as well as spatial statistical analyses of demographic data in order to measure anthropogenic change between 1984 and 2013—a socio-politically important period in Colombia’s armed conflict. We also analyze spatiotemporal relationships between socioeconomic and anthropogenic changes, which are caused by forced migration. We found that forced migration is significantly and positively related to an increasing rural-urban type of migration which results from armed conflict. Results also show that it is negatively related to interregional displacement. Indeed, anthropogenic change pertaining to different regions have had different correlations with forced migration, and across different time periods. Findings are used to discuss how socioeconomic and political phenomena such as armed conflict can have complex effects on the dynamics of anthropogenic and ecological change as well as movement of humans in countries like Colombia.

## 1. Introduction

Urban population growth across the world is one of the most influential phenomena affecting earth's sustainability and overall global change as it affects not only societies, but the environment and climate as well [1–3]. Since a myriad of definitions for urban and rural classifications exists in the literature [4] it is important to define such concepts. In Colombia, urban areas are defined according to increased infrastructure, building and transportation network density, and levels of public services, while rural areas are characterized by lower housing densities, a lack of infrastructure, and a predominance of agricultural, pasture and forest dominated land uses [5]. Although there is no official definition of peri-urban areas, these are transitional areas between rural dominated land use and covers such as forests, shrublands, pastures, agricultural areas and the previously mentioned urban areas [6]. Accordingly, in this rural, peri-urban to urban gradient, one of the key factors behind anthropogenic change is urbanization which has been documented as being one of the most influential forces in creating novel ecosystems and their respective plant and animal assemblages [7, 8]. Socioeconomically, anthropogenic change is the main cause of several contemporary epidemiological transitions as well [9] and has been a result of, and influenced by, changes in land use and economic systems [10, 11]. Indeed, the transition of a society as part of its industrialization and post-industrialization processes, has driven these modern globalized economic shifts and affected the dynamics of labor, land, and capital and the eventual reduction in the demand for agricultural labor [12, 13].

The above literature describing urbanization and global change phenomena has been well published in North America and Europe; however, there is less information explaining urbanization and industrialization phenomena in other regions of the world particularly in low and middle income countries [10, 14]. Additionally, socioeconomic factors are often key reasons for the migration of people across regions and boundaries; a fact which affects these phenomena as well. Furthermore, social and political instability, and particularly armed conflicts both internal and external to a state, can also cause a breakdown in governability and can subsequently trigger rural-urban migrations [15]. Other factors such as environmental or political events have been reported to play a lesser role in urbanization in many regions [16].

Past and current socio-political contexts in a country such as Colombia present a unique opportunity to explore these effects of migration on urbanization in a country which has one of the world’s largest documented populations of Internally Displaced People (IDP). Accordingly, by using the evolution of Colombia's armed conflict and its influence on rural-urban migration and urbanization patterns, we can better understand these dynamics in low and middle income countries. The increased availability of remote sensing techniques, geospatial data, and other information on the migration and violence caused by armed conflict in Colombia can also be used to explore the association between anthropogenic change and internal migration. Below, we propose how the Colombian context and this approach can be used to study the dynamics of urbanization and rural-urban migration of people in a novel manner [17–19].

### Colombia and its armed conflict

Armed conflict in Colombia during the past 50 years has experienced dramatic shifts and has been affected by complex socio-political phenomena that include: changes to national security policies, the influence of illicit drug groups, and globalization among many other factors that have had different impacts on its civilian society [20]. For example, between 1968 and 1982, the Armed Revolutionary Forces of Colombia (FARC) were one of many insurgent, guerilla groups. But by the end of the 1980s, Colombia had an active and complex armed conflict on multiple fronts and by this time the FARC had progressively become one of the most important insurgencies (1.200 combatants) due to several reasons [20]. First, the end of peace dialogues in 1986 strengthened the FARC as an armed insurgency and triggered the creation of other guerrilla groups. Secondly, the rise of several drug cartels coupled with political violence led to a crisis marked by the murders of political candidates, judges, and other members of the government, resulting in increased armed retaliation by the government. Both of these factors resulted in increased IDPs that peaked in 2002. Similarly, during this same time period, paramilitary groups such as the Auto-Defense Forces of Colombia (AUC) increased their military power and ranks to 8,000 combatants between 1998 and 2002. Thus 2002 represented a turning point in Colombia’s armed conflict beginning with the election of President Alvaro Uribe whose main strategy was "Democratic Security"; a government policy focused on defeating these insurgent groups through increased military pressure and by the increasing defense expenditures [21]. This increase in the conflict resulted in numerous human rights violations and IDPs [22]. Thus, the period between 1984 and 2013 presents an interesting time period to study the dynamics among rural-urban migration, armed conflict, and anthropogenic change in Colombia.

### Internally Displaced People in Colombia

Studies of intraregional, or within nation, human migration caused by armed conflicts generally considers the status of such migrants as refugees while others refer to them as Internally Displaced People (IDPs) [23]. However Hathaway [24] argues that such definitions differ according to different sources of information. For example, Bennett [25] defines IDPs as, “Persons or groups of persons who have been forced to flee homes or places of habitual residence as a result of, or to avoid, in particular, the effects of the armed conflict, situations of generalized violence, violations of human rights or natural disasters”. The author’s definition also refers to migrants who do not cross internationally recognized borders. In fact, by 1982 there were 10 refugees for every displaced person, and in 2006 there were 5 displaced persons for every 2 refugees [26]. Zetter [27] also highlights the cases of Darfur, Nepal, and Colombia as examples of intra-state wars that have caused notable increases in IDPs. Since 1990, the number of victims of forced displacement—within States—has been higher than that of refugees, but the gap increased in the second decade of the 21st century [28]. Indeed, Castles [29] points out that displacement -forced or not- have shaped the structure between “North-South relationships”.

In Colombia, displacement of people due to violence has affected 90 percent of the countries’ municipalities either by expelling or by receiving these populations [12]. Overall, IDPs are in a greater degree of vulnerability due to loss of land, homes, and employment opportunities [30]. By the middle of the 20th century, Colombian municipalities such as Quibdo, Sincelejo, and Florencia had IDP rates between 20–26%; and it was medium-sized cities that received these IDPs which were equivalent to 20% of their population in just a few years [31]. Conversely, some of Colombia’s municipalities lost more than 50% of their population due to forced migration and 10% lost nearly 25% of their population [32].

Overall, the main factors behind IDPs are their exposure to most types of violent events which results in the expulsion of rural populations from areas of conflict; as this is often a tactic used by armed groups to achieve objectives such as acquiring land tenure and natural resources [33, 34]. For example, Colombian paramilitary forces such as the AUC alone caused between 57% and 63% of recent IDPs, while Guerrilla groups caused 12 to 13%, and the remaining was caused by other unidentified groups including the State [35]. The lack of governability, or the absence of the state, its institutions, services, and security they provide, are also factors that contribute to the migrations of IDPs [36]. Studies such as those of Carrillo [37] have documented how IDPs are associated with illicit logging, illegal crops, or increased cattle ranching; activities that have all been associated with ecological changes such as deforestation.

### Remote sensing

As indicated above, the dynamics between armed conflict and IDP dynamics across space and time are complex phenomena that can be very difficult to analyze. However, the advent of remote sensing technologies and availability of socio-political data can now provide the opportunity to monitor land use-land cover and ecological changes, such as urbanization, but they can also be used to better understand the spatial dynamics of anthropogenic phenomena across spatiotemporal scales [38]. For example, anthropogenic change has been described using the interaction between greenness and multiple socio-demographic variables [38, 39] including: residential desirability and social structure [40, 41], poverty and inequality [42, 43], well-being [38, 44], and socio-demographic spatial distributions [45, 46]. Such use of remote sensing and empirical models can also be used to describe socio-economic and ecological phenomena [38, 47]. Specifically, the increased availability of geospatial data platforms, satellite imagery and increased resolution has facilitated the study of processes such as urbanization, land-use and land-cover change, deforestation, population densities, crime, housing markets, and many other problems associated with urban and regional planning [38, 48–52].

For example, high resolution images have been used to detect temporal urban changes at the scale of individual buildings, while combinations of Landsat, SPOT-5 and Synthetic Aperture Radar images have been used for single and comparative studies of deforestation and regional urban growth [38, 51, 53–55]. Night-time satellite imagery has also been frequently used to study urban and peri-urban growth and associated human-building densities, economic activities and pollution dynamics [38, 53–57]. Thus, remote sensing can be used to better measure, monitor and understand armed conflict and the movement of IDPs particularly in inaccessible, dangerous areas lacking data [58].

### Study aims and objectives

In this study we lay out an approach which uses available data and geospatial platforms to better understand how conflict, economies, and demographics drive the movement of IDP to cities given the context of the armed conflict and urbanization dynamics in Colombia [59]. As such, our aim is to explore the relationship and dynamics among armed conflict, IDPs, and land use and cover change as well as other anthropogenic, socioeconomic and ecological changes. Accordingly, using Colombia as a case study, our objectives are two-fold. First, we use remote sensing to measure national-level anthropogenic change during 1984 and 2008, a socio-politically important period in Colombia’s armed conflict. Second, we analyze how the spatial dynamics between anthropogenic change and IDPs are driven by conflict and other socioeconomic factors. We then discuss how the inter-relationships among armed conflict, movement of IDP, and anthropogenic changes can impact land use planning, public health systems, and ecological change. Such an approach can be used to better understand the political, demographic and economic drivers of anthropogenic changes on land cover and their influence on forced migration and demographic changes in countries experiencing armed conflict.

## Materials and methods

To address our objectives, we developed a systematic methodology that first characterizes land use-cover changes across space and time and that also controls for different sociodemographic variables. Second, the integrated method also accounts for different geographic scales as both armed conflict and land-use changes respond to different national, regional or metropolitan level influences. And third, uses context-specific, politically relevant time-intervals to better understand the relationship between land use-cover and IDPs and that considers delayed interactions between the variables of interest. Below, we systematically describe how we conceptually and technically address these aspects and apply our remote sensing and statistical analyses. More specific and detailed descriptions of our methods are provided in S1 File.

### Study area

Colombia is located in northern South America and in 2018 had a population of around 50 million inhabitants that are concentrated mostly in the western and northeastern areas of the country. Seven of its metropolitan areas exceed 1 million inhabitants and over 70% of its population lives in urban areas. Its geographical extension is 1,142,748 km2 (more than 4 times the size of the United Kingdom), which means a population density of 44 inhabitants per square kilometer [60].

**Fig 1:**
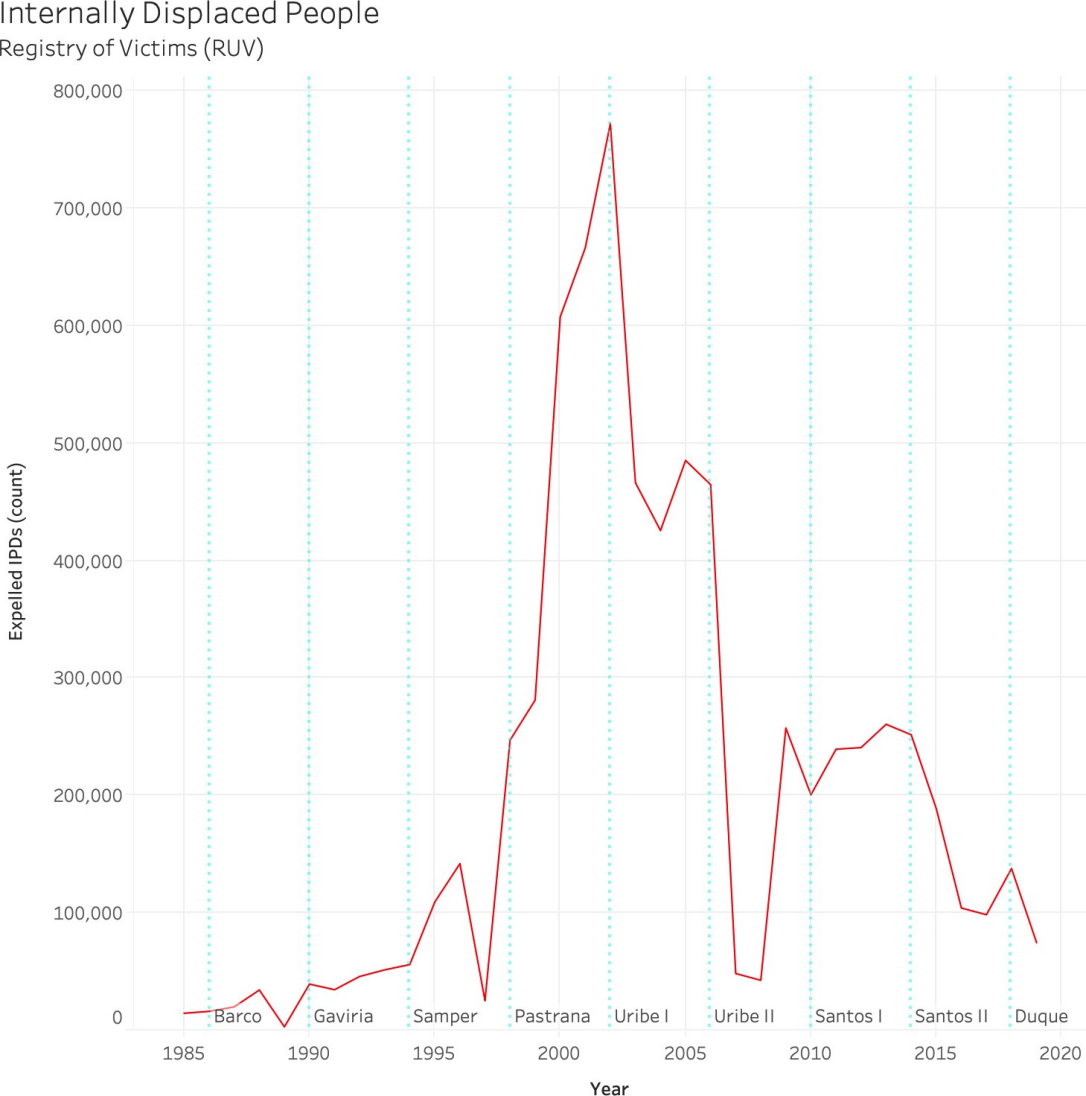
Internally Displaced People (IDP) by year. Presidential periods are shown in light blue and indicated by the president's last name.

According to Ocampo [30] between 1993 and 2005, the population of Colombia’s municipal capitals grew at a rate of 2% per year, while the rural population decreased at the rate of 0.09%. This reduction in population is associated with decreased fertility rates as well as factors associated with adolescent fertility and an increase in migration to cities by younger demographics [61]. The influence of the armed conflict on overall employment opportunities and this growth is however less known [61]. Fig 1 summarizes the relevant background information in our introduction section regarding the recent history and key dates associated with Colombia’s armed conflict and displacement of peoples. According to Colombia’s legislative framework [30], the IDPs in the country impacts both *expelling regions (i*.*e*., *expulsion)*, which are the places that the victims in question leave, as well as *receiving regions* which refer to the areas where they eventually settle.

### Geospatial and armed conflict data

We used two types of data for our analyses. We first used remote sensing data, specifically nightlight images from the Defense Meteorological Satellite Program (DMSP)/ Operational Line-Scan System (OLS) satellite “avg_vis” annual band data from 1991 to 2013 that corresponded to all available images in Google Earth Engine open source data-set platform. This data was specifically used to analyze land use-cover changes across time and to develop three indicators of land use land cover change based on night-time changes: average anthropogenic change (AAC), Anthropogenic print spatial expansion (ApSE), and Anthropogenic print spatial contraction (ApSC) which we will describe a later section below. Anthropogenic print as used hereafter, refers to changes in nighttime satellite imagery and hence is used to measure anthropogenic change and urbanization.

Secondly, we used municipal level spatial data on forced displacements for both expulsed and received populations from Colombia’s Single Registry of Victims [62] for 1984 to 2016. This dataset has been previously used in other studies of IDPS and forced migrations [63, 64]. We then used these data to develop an index of Force Migration Flows (FMF), which is the proportion of municipal population growth due to IDP caused by the armed conflict, and that takes into account both expulsion and received population (Eq 1):

$${FMF}_{T}=\frac{\sum_{t=1}^{8}Received\;IDP-\sum_{t=1}^{8}Expulsed\;IDP}{{Totalpopulation}_{t8}}$$ (1)

Additionally, we included other variables such as the demographic bonus which is a rate between each municipality’s economically active and dependent population and the percentage of urban population for each time period using 1990 to 2016 data from the Colombian National Administrative Department of Statistics (DANE). Deforestation data was obtained using Hansen et al. 's [65] Global Forest Watch (GFW) December 2013 data as well the distance of each municipality to the nearest departmental capital using the Euclidean distance raster tool in ArcGIS Desktop 10.6 software. Table 1 shows the summary statistics of the variables used in our analysis.

**Table 1:** Summary statistics of all the variables used in the statistical analysis.

Variable	Obs	Mean	Std. Dev.	Min	Max	Description
AAC T_1_	1120	0.38	1.27	-5.59	11.56	Average change in nightlight level
AAC T_2_	1120	-0.98	0.85	-9.12	5.61	
AAC T_3_	1120	2.12	1.42	0.35	11.76	
AAC T_General_	1120	1.88	2.30	-5.88	23.66	
ApSE T_1_	1120	16.9%	21.1%	0.0%	100.0%	Average growth of municipality's urban footprint
ApSE T_2_	1120	42.8%	22.1%	0.0%	100.0%	
ApSE T_3_	1120	39.0%	26.8%	0.2%	100.0%	
ApSE T_General_	1120	23.4%	26.7%	0.0%	100.0%	
ApSC T_1_	1120	9.9%	9.4%	0.0%	74.1%	Average contraction of municipality's urban footprint
ApSC T_2_	1120	1.0%	5.2%	0.0%	97.3%	
ApSC T_3_	1120	0.2%	0.9%	0.0%	12.2%	
ApSC T_General_	1120	0.3%	1.4%	0.0%	12.7%	
Forced Migration Flow (FMF): 1992–1998	1103	-2.4%	8.2%	-160.6%	15.8%	Percentage contraction of municipality's urban footprint
Forced Migration Flow (FMF): 1999–2006	1117	-9.7%	23.8%	-241.1%	45.0%	
Forced Migration Flow (FMF): 2006–2013	1120	-3.5%	8.8%	-62.6%	24.1%	
Forced Migration Flow (FMF): 1991–2013	1120	-14.7%	32.9%	-326.4%	58.8%	
Demographic Bonus (DB)	1111	0.72	0.12	0.33	1.46	Ratio between active and dependent population
% of urban population in 1992	1047	0.36	0.23	0.00	1.00	Percentage of municipal population who live in the urban area
% of urban population in 1999	1103	0.39	0.24	0.00	1.00	
% of urban population in 2000	1112	0.39	0.24	0.00	1.00	
% of urban population in 2007	1117	0.42	0.24	0.00	1.00	
% of urban population in 2017	1120	0.44	0.25	0.00	1.00	
Distance to a Capital City	1117	61.34	43.95	1.00	414.56	Distance in kilometers to departmental capital
% of municipal area with forest loss: 2000–2017	1120	0.03	0.04	0.00	0.26	Percentage of deforested municipal area

AAC is average anthropogenic change, ApSE is Anthropogenic print spatial expansion, and ApSC is Anthropogenic print spatial contraction (ApSC). T1, T2 and T3 are the first (1991–1998), second (1999–2006), and third (2006 to 2013), analyzed time periods respectively.

### Remote sensing analyses

One of our objectives was to analyze the relationship between changes in forced displacement (i.e., IDPs) and land use-cover. To do so, we used nightlight images from DMSP/OLS satellite data and measured three time periods (1991 to 1998, 1999 to 2006, and 2006 to 2013) and changes in nightlight intensity (average anthropogenic change: AAC) and in the geographical extension of areas with high light intensity. For this first analysis we used Google Earth Engine and the 3 time periods as they are related to relevant changes in Colombia’s armed conflict. The first period of 1991 to 1998 was characterized by the intensification of violence and a notable increase in IDPs (see Fig 1). The second period of 1999 to 2006 was marked by President Alvaro Uribe and a concerted military response known as “Democratic Security” by the Colombian government, which generated a significant decrease of forced migration flows while at the same time experiencing the largest absolute numbers of IDPs. Finally, the third 2006 to 2013 period during the government of President Juan Manual Santos initiated the peace process with the FARC.

For the nightlight intensity change analysis, we used ArcGIS Desktop 10.6, to develop two additional variables to measure geographic changes in nightlight extension: Anthropogenic print spatial expansion (ApSE) which measures the proportion in which high light intensity areas had expanded and Anthropogenic print spatial contraction (ApSC), which measures—if present- the proportion of area that stops emitting light during each period. In our subsequent statistical analysis, we focus on ApSE in subsequent analyses since it allows us to better analyze landscape changes such as urbanization which is key in meeting our study objectives. For more specific details concerning the development of these indicators, please see S1 File, section 1: *Detailed remote sensing analysis*.

### Statistical modeling

We used ApSE and FMF to develop a three step statistical analysis to test our study objective that forced migration due to armed conflict can significantly affect land use-cover changes. First, we integrated and controlled for other potential factors that could be related to land use-cover changes. Second, we used several statistical models, explained below, to identify the geographic heterogeneity of the relationship between forced displacement and land use-cover changes across different regions and metropolitan areas. And finally, we used different temporal specifications of forced migration flows in the form of temporal lags to study the short, middle, and long-term effect of displacement over land use-cover changes (See S3 Fig for more detailed methods in S1 File). Accordingly, the basic structure of our models is shown in Eq 2.

$${ApSE}_{it}=\alpha +\beta (FMF_{il})+\theta (Dem_{it}^{\prime })+\varphi (Ge{o\prime }_{i})+e_{it}$$ (2)

where the anthropogenic print’s expansion (ApSE) in a municipality “*i”* in a period “*t*”, is determined by the forced migration flows “*FMF*” calculated with different forms of a lag “*l*”. Additionally, we control by a vector “*DEM’*” of demographic variables such as the demographic bonus and its quadratic terms and the percentage of population which lives in urban areas. Another vector of geographic variables “*Geo’*”, contains both the logarithmic distance from each municipality to its departmental capital and the percent of municipal area that has been deforested. This Eq (2) was used to better understand the relationship between landscape changes and force migration dynamics while controlling for other key factors.

All statistical analyses and models were based on cross-sectional data at the municipal level for each of the 3 time periods. Specifically, we used an Ordinary Least Squares (OLS) model to analyze the relationship between forced displacement and land-use changes at a national level. We also ran a Durbin spatial error model [66] (S5 and S6 Equations in S1 File) and a Durbin model for spatial lag (S7 Equation in S1 File) to study the possible spatial interaction between FMF and ApSE. All these analyses for both the spatial lag and spatial error models were performed using the “spatreg” package with STATA 14. We also tested for the robustness of our results as described in detail in S1 File, to study the heterogeneous effects of forced displacement across different spatial regions and metropolitan areas, we used a Geographically Weighted Regression (GWR) (S8 and S9 Equations in S1 File). The GWR analysis and the spatial matrix needed for the other spatial regressions were done using ArcGIS Desktop 10.6. A detailed description of these models is provided in S1 File (S8 and S9 Equations).

## Results

In this section we present our results in the following order. First, national level results are presented from the OLS robust, spatial lag and spatial error models. Then we briefly describe the results obtained by our GWR analysis at a regional and local scale for seven main metropolitan areas in Colombia: Bogota, Aburra Valley, Cali, Barranquilla, Cartagena, Bucaramanga, and Cucuta.

We found that the percentage of Urban Population variable is positive and statistically significant in all estimated models at the national level. This result indicates that as the urban area (percentage of the total area of the municipality) increases in the initial period, urban expansion also increases. This effect was stronger for the regression models for the period 1991–1998. A 1% increase in the urban area at the beginning of this period results in an increase of 0.18% in urban expansion. The negative coefficient of Distance to a Capital City indicates that the proximity to a large urban center is associated with increased urban expansion. This can be explained by the strong economic interactions that may occur between large urban nuclei and the surrounding municipalities that may trigger urban growth [67]. We also test alternative specifications using additional demographic variables such as demographic growth and estimate internal non forced migration to check for robustness (see S1 File, section III: *Additional statistical tests*).

We found that FMF, our variable of interest, was positively and statistically significant in relation to anthropogenic urban expansion. However, the variable is significant in the OLS and Spatial Lag models, but not in the Spatial Error Model (Table 2). Overall, the magnitude of parameter estimates is greater in the OLS model than in the Spatial Lag version. We also found that the magnitude of the coefficients decreases as the time period for the estimate increases. For example, in the Spatial Lag model the coefficient, in the period 1991–1998 the coefficient of FMF is 0.650, while the value for the same variable in period 2006–2013 is 0.086.

**Table 2:** Anthropogenic print Spatial Expansion (ApSE) regression analysis with one lag over Forced Migration Flows (FMF).

Independent Variables	OLS Robust Regression Model			Spatial Lag Regression			Spatial Error Regression		
	T1	T2	T3	T1	T2	T3	T1	T2	T3
	1991–1998	1999–2006	2006–2013	1991–1998	1999–2006	2006–2013	1991–1998	1999–2006	2006–2013
Forced Migration Flow (FMF)	1.540***	0.278***	0.160***	0.650**	0.119**	0.086***	0.544	0.109	0.085***
	(0.362)	(0.093)	(0.034)	(0.281)	(0.058)	(0.018)	(0.34)	(0.067)	(0.022)
Demographic Bonus	-1.708***	0.549	-1.110**	-1.192***	-0.026	-0.596**	-1.527***	-0.35	-0.737**
	(0.38)	(0.357)	(0.472)	(0.237)	(0.261)	(0.243)	(0.316)	(0.348)	(0.327)
Demographic Bonus Squared	0.826***	-0.363*	0.427	0.640***	0.006	0.315**	0.818***	0.18	0.428**
	(0.219)	(0.213)	(0.276)	(0.15)	(0.166)	(0.154)	(0.196)	(0.216)	(0.203)
% of urban population	0.250***	0.155***	0.132***	0.181***	0.089***	0.107***	0.172***	0.071***	0.110***
	(0.032)	(0.031)	(0.034)	(0.02)	(0.022)	(0.02)	(0.025)	(0.027)	(0.024)
Distance to a Capital City (km)	-0.034***	-0.060***	-0.069***	-0.012**	-0.018***	-0.012**	-0.006	-0.006	0.005
	(0.01)	(0.011)	(0.013)	(0.005)	(0.006)	(0.005)	(0.007)	(0.007)	(0.007)
% of municipal area with forest loss	0.015	-0.088	-0.038	-0.028	0.062	0.118	0.171	0.155	0.428**
	(0.157)	(0.194)	(0.187)	(0.13)	(0.141)	(0.132)	(0.177)	(0.195)	(0.186)
*RHO*				0.695***	0.767***	0.846***			
				(0.025)	(0.023)	(0.017)			
*Lamda*							0.753***	0.790***	0.886***
							(0.026)	(0.023)	(0.016)
Constant	1.004***	0.405***	1.190***	0.558***	0.153	0.331***	0.812***	0.601***	0.693***
	-0.158	-0.148	-0.193	-0.094	-0.102	-0.097	-0.127	-0.14	-0.136
Observations	1,041	1,096	1,109	1,041	1,041	1,041	1,041	1,041	1,041
R-squared	0.343	0.147	0.259	0.633	0.595	0.757	0.324	0.102	0.137
AIC	-704.6	-374.8	-99.3	-1171	-927.1	-1020	-1103	-895.2	-961.7
BIC	-670	-339.8	-64.2	-1127	-882.5	-975.1	-1058	-850.7	-917.2
Log-likelihood	359.3	194.4	56.63	594.7	472.5	518.8	560.3	456.6	489.9

Note, T1 is Time period 1 or 1991–1998; T2 is Time period 2 or 1999 to 2006; T3 is Time period 3 or 2006–2013.

*** p- value < 0.01

** p-value < 0.05

*p-value < 0.10. Standard error in parenthesis. AIC: Akaike Information Criterion. BIC: Bayesian information criterion.

According to goodness of fit, the Spatial Lag model best fits our data. Overall, the R2 statistic is higher in the spatial lag model (0.757 for T3) compared to the R2 of the OLS (0.259) or the Spatial Error models (0.137). The best goodness of fit of the Spatial Lag model is also indicated by its higher values for the Log-likelihood for this specification when compared to the Log-likelihood of the OLS and Spatial Error models (Table 2). Using the selected Spatial Lag model as a reference, we explored the effect of FMF using different temporal lags, namely: one lag, half a lag, and no lag (Table 3).

**Table 3:** Anthropogenic print Spatial Expansion (ApSE) and Forced Migration Flows (FMF) using the spatial lag regression output for 3 time periods T1, T2 and T3.

	One lag over FMF			Half lag over FMF			No lag over FMF		
Independent Variables	T1	T2	T3	T1	T2	T3	T1	T2	T3
	1991–1998	1999–2006	2006–2013	1991–1998	1999–2006	2006–2013	1991–1998	1999–2006	2006–2013
Forced Migration Flow (FMF)	0.650**	0.119**	0.086***	0.293*	0.032	0.199***	0.076	0.036*	0.260***
	((0.281)	((0.058)	(0.018)	(0.163)	(0.021)	(0.035)	(0.052)	(0.02)	(0.056)
Demographic Bonus	-1.192***	-0.026	-0.596**	-1.195***	-0.035	-0.631***	-1.213***	-0.037	-0.711***
	(0.237)	(0.261)	(0.243)	(0.238)	(0.261)	(0.242)	(0.237)	(0.261)	(0.243)
Demographic Bonus Squared	0.640***	0.006	0.315**	0.641***	0.013	0.345**	0.653***	0.016	0.398**
	(0.15)	(0.166)	(0.154)	(0.15)	(0.166)	(0.154)	(0.15)	(0.166)	(0.155)
% of urban population	0.181***	0.089***	0.107***	0.183***	0.089***	0.097***	0.185***	0.087***	0.097***
	(0.02)	(0.022)	(0.02)	(0.02)	(0.022)	(0.02)	(0.02)	(0.022)	(0.02)
Distance to a Capital City (km)	-0.012**	-0.018***	-0.012**	-0.012**	-0.018***	-0.010*	-0.012**	-0.018***	-0.010*
	(0.005)	(0.006)	(0.005)	(0.005)	(0.006)	(0.005)	(0.005)	(0.006)	(0.005)
% of municipal area with forest loss	-0.028	0.062	0.118	-0.06	0.048	0.16	-0.072	0.058	0.114
	(0.13)	(0.141)	(0.132)	(0.128)	(0.141)	(0.132)	(0.128)	(0.142)	(0.132)
*RHO*	0.695***	0.767***	0.846***	0.698***	0.769***	0.843***	0.699***	0.768***	0.846***
	(0.025)	(0.023)	(0.017)	(0.025)	(0.023)	(0.018)	(0.025)	(0.023)	(0.017)
Constant	0.558***	0.153	0.331***	0.558***	0.157	0.341***	0.564***	0.156	0.368***
	(0.094)	(0.102)	(0.097)	(0.094)	(0.102)	(0.096)	(0.094)	(0.102)	(0.097)
Observations	1,041	1,041	1,041	1,041	1,041	1,041	1,041	1,041	1,041
R-squared	0.633	0.595	0.757	0.633	0.595	0.759	0.633	0.596	0.757
AIC	-1171	-927.1	-1020	-1169	-925.1	-1029	-1168	-926	-1020
BIC	-1127	-882.5	-975.1	-1125	-880.5	-984.7	-1124	-881.4	-975.1
Log-likelihood	594.7	472.5	518.8	593.6	471.5	523.6	593	472	518.8

Note, T1 is Time period 1 or 1991–1998; T2 is Time period 2 or 1999 to 2006; T3 is Time period 3 or 2006–2013.

*** p- value < 0.01

** p-value < 0.05

*p-value < 0.10. Standard error in parenthesis. AIC: Akaike Information Criterion. BIC: Bayesian information criterion.

We estimated the Spatial Lag on our models for the periods T1, T2 and T3. Overall, the results of these models corroborate that the Spatial Lag model with a temporal lag of one period presents the strongest effect of FMF. For the period 1991–1998 (T1), the positive coefficient of 0.650 (6.5% increase in urban expansion) that accompanies the variable FMF indicates a 10% increase in the migration flow. When we reduce the temporal lags to half lag and no lag, the effect of forced migration on urban expansion dissipates with the exception of the period T3 = 2006–2013, where the instantaneous effect of Flow Migration seems stronger (0.26 vs 0.19 in the half lag model and 0.086 in the one lag model). In all cases, the coefficient Rho for the Spatial Lag variable is positive and statistically significant at all levels.

### Geographic distribution of the interaction between ApSE and FMF: Regional and metropolitan interactions

**Fig 2:**
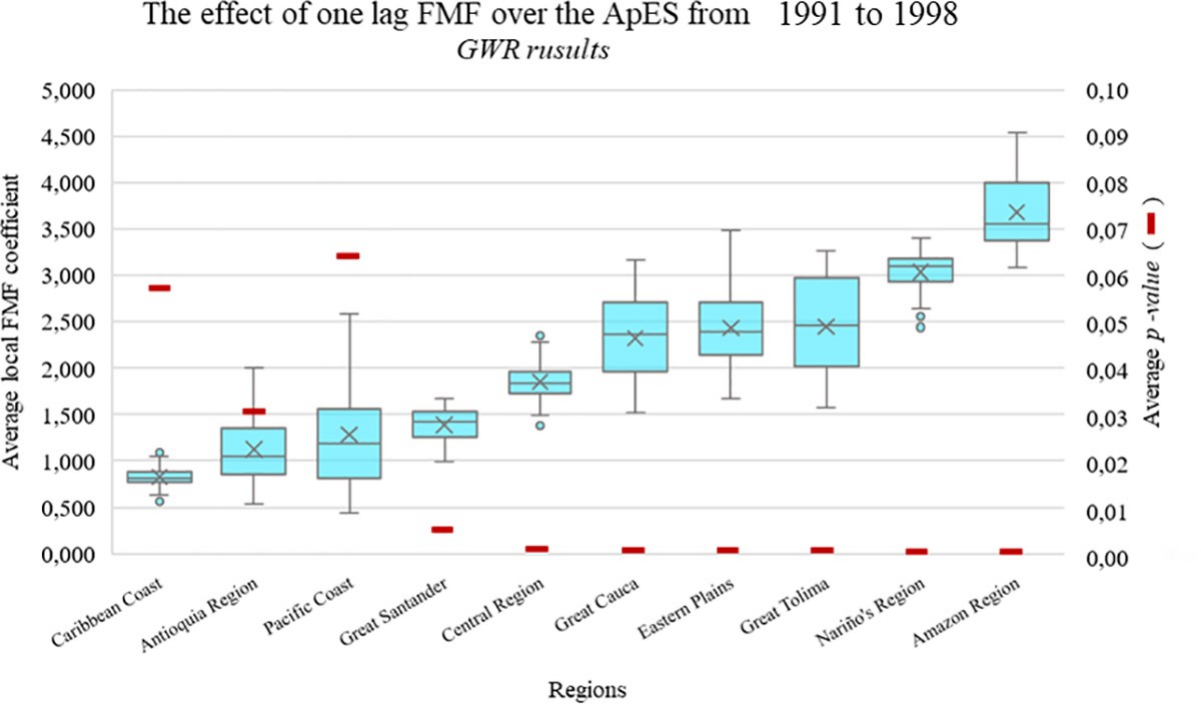
GWR (Geographically Weighted Regression) detected coefficients of one lag FMF (Forced Migration Flow) over the ApES (Anthropogenic print Spatial Expansion) from 1991 to 1998.

All regional-level GWR models were analyzed using a sample of 1,041 municipalities. Fig 2 shows the distribution of the coefficient of the FMF between 1984 and 1991 over the ApSE from 1991 to 1998. The graph shows the relationship between FMF from the 1980’s and the ApSE in the 1990’s and differentiated according to region. Note that the effect of violence from the 1980’s, had significant effect over the configuration of the ApSE in the 1990’s across all the regions (p-value < 0.1). The correlation between ApSE and FMF was stronger in some regions than others. For example, in the Eastern Plains and the Great Tolima regions, the effect of violence on the ApSE was 2.2 times higher than in the Antioquia region (where cities like Medellin, Pereira or Armenia are located).

We also found that AsPE in the 1990’s for both the Pacific and Caribbean coasts of Colombia were less related to FMF than inland territories and thus, there seems to be a national-level periphery-center behavior with respect to violence and urban expansion. In the Nariño and Amazon regions we found that the effect of the FMF over the ApSE was significantly higher than in the rest of the country (*p-value* < 0.01*) (see S2 Table* in S1 File*)*.

**Fig 3:**
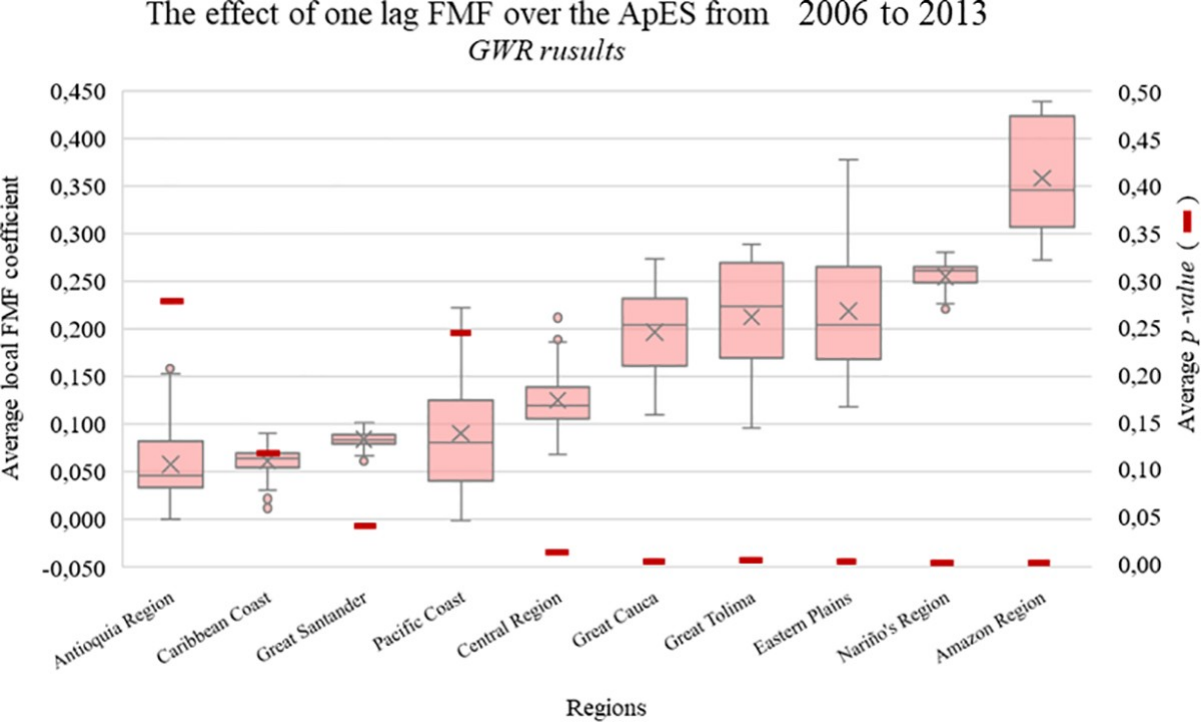
GWR (Geographically Weighted Regression) detected coefficients of one lag FMF (Forced Migration Flow) over the ApES (Anthropogenic print Spatial Expansion) from 2006 to 2016.

Several key differences were identified in the relationship between FMF in the 1990s and the ApSE from 1999 to 2006 (S5 Fig in S1 File). In particular, the loss in the significance of FMF in five of the 10 regions was notable. This, however, does not suggest that there was no expansion of the anthropogenic print in these regions or forced migration. In fact, the Antioquia region experienced the largest proportional expansion. But rather, there seems to be no significant relationship between these two variables for half of the regions within this time period. We also observed a loss in significance of FMF effect over the anthropogenic print for both Antioquia and the central regions of Colombia. Our results also indicate the relationship between FMF and ApSE over the last analyzed time period as an instance where FMF recovers predictive ability towards anthropogenic change compared with T2 (Fig 3). For additional tables and figures with the detailed results from the GWR analysis, see S1 to S3 Tables and S4 Fig in S1 File.

**Fig 4:**
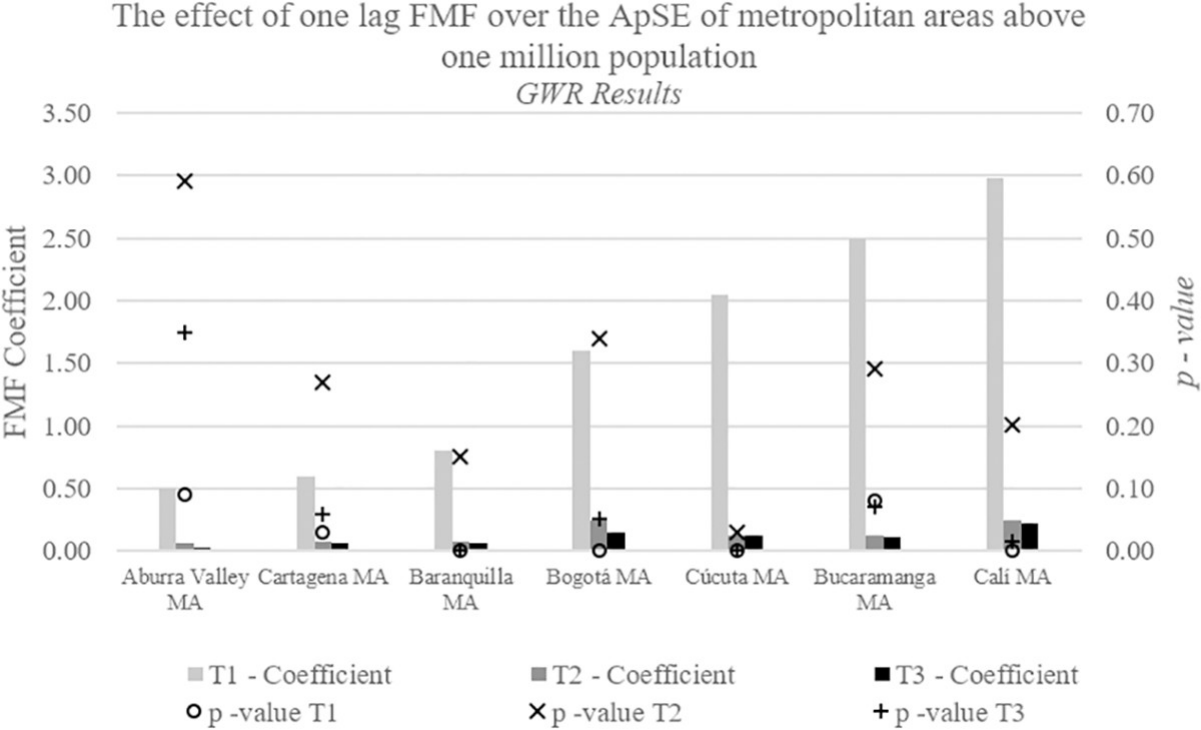
GWR (Geographically Weighted Regression) detected coefficients of one lag FMF (Forced Migration Flow) over the ApES (Anthropogenic print Spatial Expansion) of metropolitan areas above one million inhabitants. Note, T1 is Time period 1 or 1991–1998; T2 is Time period 2 or 1999 to 2006; T3 is Time period 3 or 2006–2013.

More than 20.2 million people live in the seven analyzed metropolitan areas (MA), accounting for 45% of Colombia’s population (according to the 2018 national Census). Results in Fig 4 suggest that there is little effect of FMF on ApSE in the MAs or that it might not have been adequately captured by our model, particularly during T2. The T1 is significant and shows a positive effect with coefficients above 0.5 for most MAs, particularly those close to Venezuela, except for the Aburra Valley MA (i.e., Medellin). Thus confirming the observed regional effects in previous models.

## Discussion

This study explored spatiotemporal relationships between IDPs, anthropogenic, and demographic changes in Colombia at different scales. Our integrated approach using remote sensing and geospatial data platforms–while controlling for both spatial autocorrelation and temporal effects—analyzed national-level anthropogenic change over a period of 20 years marked by one of the most intense armed conflicts in the world. We also explored relationships at metropolitan-level dynamics to better assess our results. Findings indicate that the effect of lagged FMF on ApSE was stronger in the period before the armed conflict reached its peak in terms of IDPs (T1 = 1991–1998) that was preceded by violent guerrilla and drug trafficking activity (Lag T1 = 1984–1991). Furthermore, our results suggest a strong spatial interaction among neighboring municipalities in the process of urban expansion as indicated by both increasing ApSE of neighboring municipalities and the observed municipality; suggesting that municipal administrative units are not the most appropriate scale to observe the expansion of anthropogenic change, and thus the association between FMF and ApSE should be broadly construed as a more regional-level phenomenon.

Our findings are consistent with other studies [68–70]. First, our results suggest that both migration and demographic structure play an important role in urban growth, and most interestingly, and that neither is a predominant force driving anthropogenic change over time. We found that FMF had a stronger effect on ApSE when it peaked (1999–2006) than in previous periods (Tables 2 and 3). However, we also found evidence that population structure is strongly associated with ApSE over periods of less intense forced migration (Tables 2 and 3). This increase in FMF observed during 1999–2006 rendered structural characteristics of the population less important in terms of anthropogenic change. This finding has implications for other demographic studies that aim to describe the relative importance of these variables in the context of the demographic transition. Specifically, our findings suggest that the main driver in the process of urbanization of a country can change from population structure towards migration over periods of 8 years, which is a rather short time period in terms of demographic transitions.

The FMF recovered predictive power in the last time period (T3), suggesting that the expansion of the anthropogenic print between 1999 and 2006 follows a different trend with respect to the ApSE between 1991 and 1998 and the ApSE between 2006 and 2013. Colombia’s political history associated with each of these time periods provides the context to better understand our findings. Specifically, T2 is both the peak of FMF and coincides with one of the most controversial political events during our analysis period; a public policy by a right-wing government whose aim was to regain national security; while T1 was associated with a context of violence and political instability due to increased drug-trafficking.

In terms of our control variables, our findings also identified a relationship between areas receiving IDP and their rates of deforestation [13]. Carrillo [37] suggests that the precarious conditions in which some IDPs arrive not only in peri-urban but also in rural areas force them to participate in activities such as logging, illegal crops or cattle ranching. Such factors are often associated with increased land use-cover changes and deforestation as documented by several other authors [59, 71]. Other illegal activities such as illegal drug cultivation and mining are also key activities in which IDPs participate that lead to detrimental ecological impacts and changes [72].

The migration paths taken by IDPs are also not strictly rural-urban but also rural-rural. This can take place both in the receiving and expelling areas but is more significant in the former given the population density. Overall, studies on forced migration document how IDPs experience a substantial decrease in overall well-being. Such internally displaced households experience considerable decreased aggregate consumption per adult equivalent [12], access to state services and infrastructure, and governability or a strong institutional presence. As such all improving all these factors could contribute to mitigating the incidence of IDP [36].

In terms of the regions and metropolitan areas studied, we found that there are some caveats to our findings. For example, ApSE was less related to FMF in coastal regions during the period 1991–1998, possibly as a result of stronger violence in these regions. This suggests these regions were sources of migration, while other regions were receiving (sinks) IDPs. We found a loss of significance of the effect of FMF on ApSE in Antioquia, but this does not necessarily mean that they did not experience spatial expansion of the anthropogenic print (Table 2; Figs 3 and 4; S5 Fig in S1 File). Further research is needed to determine if migration was not related to violence, but rather, due to rural poverty and living conditions, and other economic and globalization factors; thus rendering the effect of FMF insignificant.

Our study also presents some limitations. First, our findings are based on the anthropogenic print as measured using nighttime satellite imagery and this is only one of many methods available to study urban growth. Future studies using other geospatial data with greater heterogeneity and resolution are needed to increase our understanding of the relationship between migration and urban growth variables. Another limitation was that our migration dataset was also composed of aggregated values. Even though we used the official data for FMF, we verified its consistency with other data sources in S1 File, as a means of verification of our findings (see S5 and S6 Tables in S1 File). As better information becomes available over the routes of IDPs over space and time, these processes will also be better understood. A particular novel area of future research is that as mobile phone data becomes increasingly available to describe more recent mobility patterns of migrant populations and we will also be able to associate specific migrant profiles to spatial patterns. Datasets including source and destination of migrants will enable researchers to better determine, for example, the role of rural to urban migration within national mobility patterns, and their role in urbanization (our study is limited to observe the effect of urban population size). That said, our study does provide an approach and way forward in addressing a set of questions that can be better framed and analyzed based on this study’s approach.

## Conclusion

Internal migrations due to armed conflicts have been documented to affect the social fabric of many societies. We found that the migration of IDPs has not directly impacted the larger urban centers; rather, this migration has substantially impacted smaller populations (often in proximity of large urban areas). This has important implications for issues related to land use planning, public health, and even deforestation. However, migration due to violence is not a static process, but rather, a heterogeneous and complex process. The FMFs vary according to different contexts such as violence and the demographic transition of the local population, which makes formulation of public policies aimed at dealing with the effects of IDP migrating to cities very complex.

The problems associated with IDPs and their migration are not exclusive to the largest urban populations or peri-urban areas that are receiving them, although these are often the areas that have been documented as requiring the most governmental assistance [6]. Our results show that smaller urban areas experienced greater perturbations from migration processes than large urban centers, where both migrating and receiving populations require more assistance [73]. Similarly, we found that sources of IDP also undergo particular detrimental dynamics such as reduced economic activity and increased deforestation [12]. Hence, it is important to address the problems of IDP in both expelling and receiving areas.

Our study illustrates how anthropogenic change influences migration processes and shapes human landscapes in Latin American cities. Little is known about the impact of migration on human occupation of the environment, however this and many other questions related to population flows can be addressed using this type of analysis and our approach. The use of satellite imagery platforms and available geospatial data are also one of the promising and emerging technologies that can be used to explore human flows. Further studies of this phenomenon can include other data-driven methods such as machine learning in classifying images, and well as the application of diverse methodologies to analyze these types of human flow datasets.

Understanding human flows and being able to estimate them from readily available and no cost data and information can be crucial for public policy formulation and impact evaluation. Estimating flows of vulnerable populations, often undocumented, within a country is fundamental to many government responsibilities, including resource allocation, disease risk, epidemics and other public health activities and maps in the context of natural hazards and climate change, among others. Our study hopefully contributes to better understanding these processes and that it is possible to measure human flows using satellite imagery and available socioeconomic data (i.e., census, armed conflict, infrastructure) to better inform public policy.

## Supporting information

S1 File (DOCX)
