# Supplementary material for: Exploring the dynamics of migration, armed conflict, urbanization, and anthropogenic change in Colombia
Source: PLoS One. 2020 Nov 24;15(11):e0242266. doi: 10.1371/journal.pone.0242266 (PMC7685458; doi:10.1371/journal.pone.0242266)
Supplement: S1 File — (DOCX) [file pone.0242266.s001.docx]

**Supplement 1**

1. *Detailed remote sensing analysis*

We used remote sensing to address our objectives in multiple phases as shown in Figure S-1. In the first phase of our analysis we used twelve different nightlight rasters from the Defense Meteorological Program Operational Linescan System (DMSP OLS - Nighttime Lights Time Series Version 4 raster data set), as well as image and data processing by NOAA's National Geophysical Data Center. In addition, the rasters selected from DMSP OLS were taken from spectral bands containing unfiltered mean values of visible light. Figure S-1 also shows the data processing approach which was implemented to generate the following variables used in our analyses: Average Anthropogenic Change (AAC), the Anthropogenic Print Spatial Expansion (ApSE) and the Anthropogenic Print Spatial Contraction (ApSC) for Colombia from 1991 to 2013.


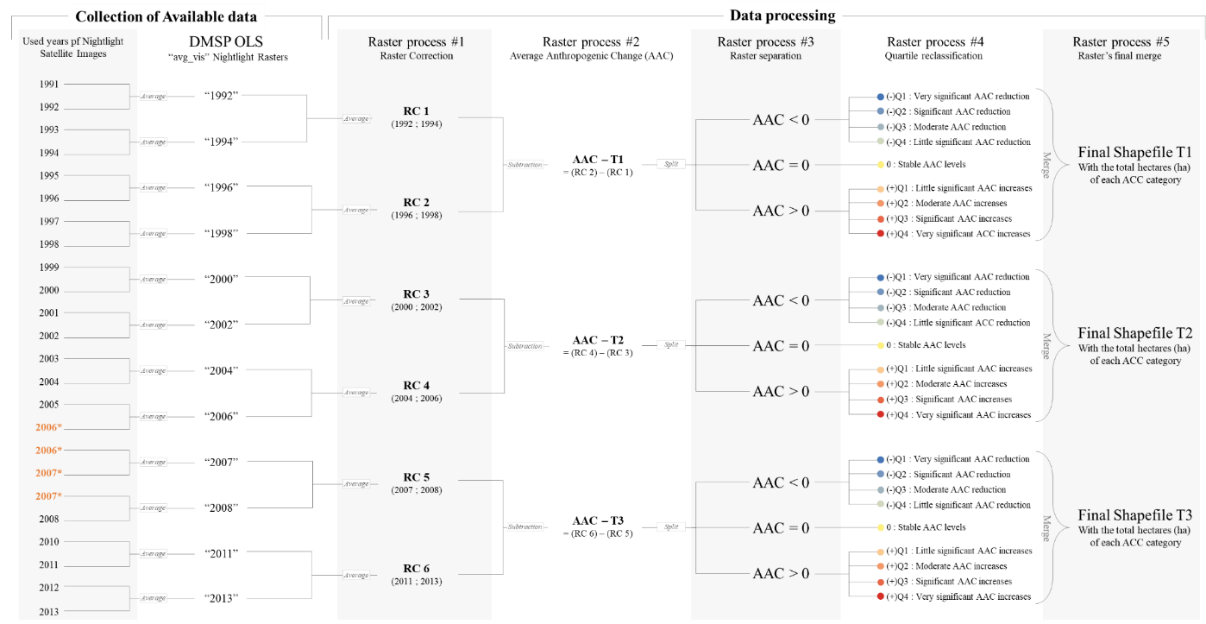


**Figure S-1.** Data processes showing the workflow of converting different raster files of satellite imagery into datasets that adequately described our main variable of interest: anthropogenic change over specific time-periods.

We measured anthropogenic change using the Nighttime Lights Time Series (Nighttime hereafter) for two reasons. First nighttime imagery has been used to previously describe population flows, as opposed to land use-cover change measured by classification of multi-spectral satellite imagery (37,52–56). Second, it was available for the whole study period, unlike other satellite imagery used to analyze urbanization processes.

For our second analysis, we used the Google Earth Engine platform and created six new corrected raster files (RC) from the average of two consecutive DMPS OLS files (raster process 1). Raster process 2 consisted of calculating the AAC using Google Earth Engine for three time periods of interest: 1991-1998, 1999-2006 and 2006-2013. With the calculated AAC raster using Equation S-1, we extracted several municipal level statistics including: average AAC's, standard deviations for the AAC's, and the AAC's maximum and minimum values.

$AAC=\Delta RC={RC}_{T+1}-{RC}_{T}=\frac{\sum_{t_{+5}}^{t_{+8}} avg\_vis}{4}-\frac{\sum_{t}^{t_{+4}} avg\_vis}{4}$ (Equation S-1)

Raster process 4, consisted of reclassifying positive and the negative values for each raster into quartiles and transforming them into a shapefile. As shown in raster process 5, we obtained a final database which classified Colombia into 9 different categories. The resulting datasets are shown in Figure S-2.


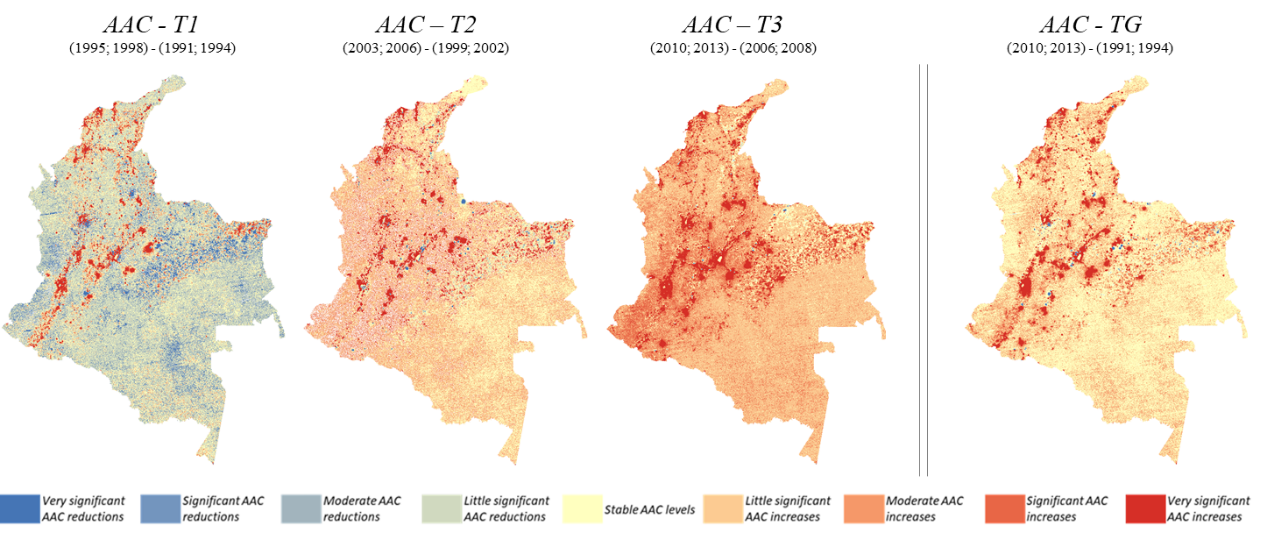


**Figure S-2.** Visual representation of the spatial dataset used for the econometric analysis using DMSP-OLS Nighttime Lights Time Series. Raw Images and Data processing by NOAA's National Geophysical Data Center. DMSP data collected by the US Air Force Weather Agency. The base map was build using the municipal open source shape file from The National Department of Statistics (DANE), which can be consulted and downloaded at the following link <https://geoportal.dane.gov.co/servicios/descarga-y-metadatos/descarga-mgn-marco-geoestadistico-nacional/>.

For each of the three time periods (i.e. T1, T2 and T3) and analyzed municipalities, we calculated the total area covered by each of the nine AAC categories. Anthropogenic Print Spatial Expansion (ApSE) was calculated by dividing the total area (in hectares) for the greatest quartiles of AAC (areas that in a specific time period experienced either significant, or very significant AAC increases) by the total hectares of each municipality. Equations S-2 and S-3 summarize both spatial indices. Overall, AAC depicts changes in nightlight intensity levels, but it does not directly reflect the geographic expansion of anthropogenic activity (such as urban expansion).

${ApSC}_{T}=\frac{{Ha}_{i}||{{(-Q1)}_{T}+(-Q2)}_{T}}{TotalHa}$ (Equation S-2)

${ApSE}_{T}=\frac{{Ha}_{i}||{{(+Q3)}_{T}+(+Q4)}_{T}}{TotalHa}$ (Equation S-3)

${FMF}_{T}= \frac{\sum_{t=1}^{8} Received population-\sum_{t=1}^{8} Expelled population}{{Total population}_{t8}}$ (Equation S-4)

These different measurements were implemented because there are some areas with large variations in AAC levels where no significant changes related to the size of the anthropogenic print were observed. Thus, increases in nightlight intensity could be related to building densification processes, electrification programs, or increases in electrical consumption. Then, we estimated the relationship between forced migration and urban expansion using “forced migration flow” (FMF) (equation S-4), that represents the percentage of population growth or the percentage of its decrease due to forced migration for each municipality. Because we used just 3 different time periods in our analysis, the opportunity to run panel base regression models was limited.

1. *Temporal lags and additional spatial econometric models*

We used different specifications within the migratory flux range (FMF), lagging this in time relative to the periods of anthropogenic change (ApSE). Thus, three types of lags were used: I) No lag (zero lag), in which the measured period of forced migration is the same measured period of anthropogenic change, to observe short-term effects; II) Medium lag (1/2 lag), which takes four years before the measured period of anthropogenic change and four years within the period of anthropogenic change, to measure medium-term effects; and III) One lag (1 lag), which takes the previous eight years of migratory flows to explain the next eight years of anthropogenic change, to measure long-term effects. The following Figure S-3 provided a graphic explanation of these lags.


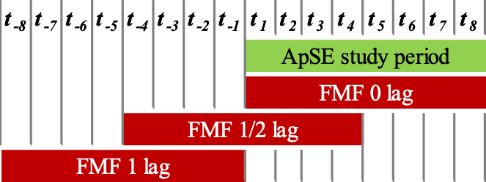


**Figure S-3**: Temporal lags over FMF variable.

The spatial error model creates a matrix of spatial weights based on the neighboring municipalities' error terms. This operation and form of modeling allows one to controll the unobservable factors of the neighboring municipalities. The mathematical representation of this model is shown in equation S-5 and S-6; to understand the basic structure of the general model, please consult the methods and material section in the article.

${ApSE}_{it}=\alpha+\beta\left( FMF_{il} \right)+\theta\left( Dem_{it}^{'} \right)+\varphi\left( Ge{o'}_{i} \right)+\varepsilon_{it}$(Equation S-5)

$\varepsilon=\rho(Wu_{it})+e_{it}$(Equation S-6)

As shown in the following equation (Equation S-7), the spatial lag model integrates a neighborhood matrix that interacts with the dependent variable. This interaction between the spatial weights of the neighbors and their respective values within the dependent variable (ApSE), enable one to understand how the anthropological change processes of neighboring municipalities affect, or are affected by the anthropological change of a municipality "i". For the construction of both the spatial lag and spatial error models, a queen-type spatial weight matrix was used (‘*W* = 1’ for all municipalities that share direct contiguity, in any direction). In these regressions, island territories were excluded.

${ApSE}_{it}=\alpha+\beta\left( FMF_{il} \right)+\theta\left( Dem_{it}^{'} \right)+\varphi\left( Ge{o'}_{i} \right)+\rho(W*ApSE_{t})+e$ (Equation S-7)

For its part, the GWR model (equations S-8 and S-9) allows for the calculation of the spatial heterogeneity of the coefficients of multivariate regression, at a given level of aggregation. In our particular case, the model was used at the municipal level, and the results summarized at the regional (se figure S-4) and metropolitan area scales. The quadratic term of the demographic bonus was excluded within this specification.

${ApSE}_{it}(u)=\alpha_{i}(u)+\beta_{1i}(u)\left( FMF_{il} \right)+\beta_{2i}(u)\left( Dem_{it}^{'} \right)+\beta_{3i}(u)\left( Ge{o'}_{i} \right)+e$ (Equation S-8)

$\beta^{^}\left( u \right)=\left( X^{T}W\left( u \right)X \right)^{-1}X^{T}W\left( u \right)y$ (Equation S-9)


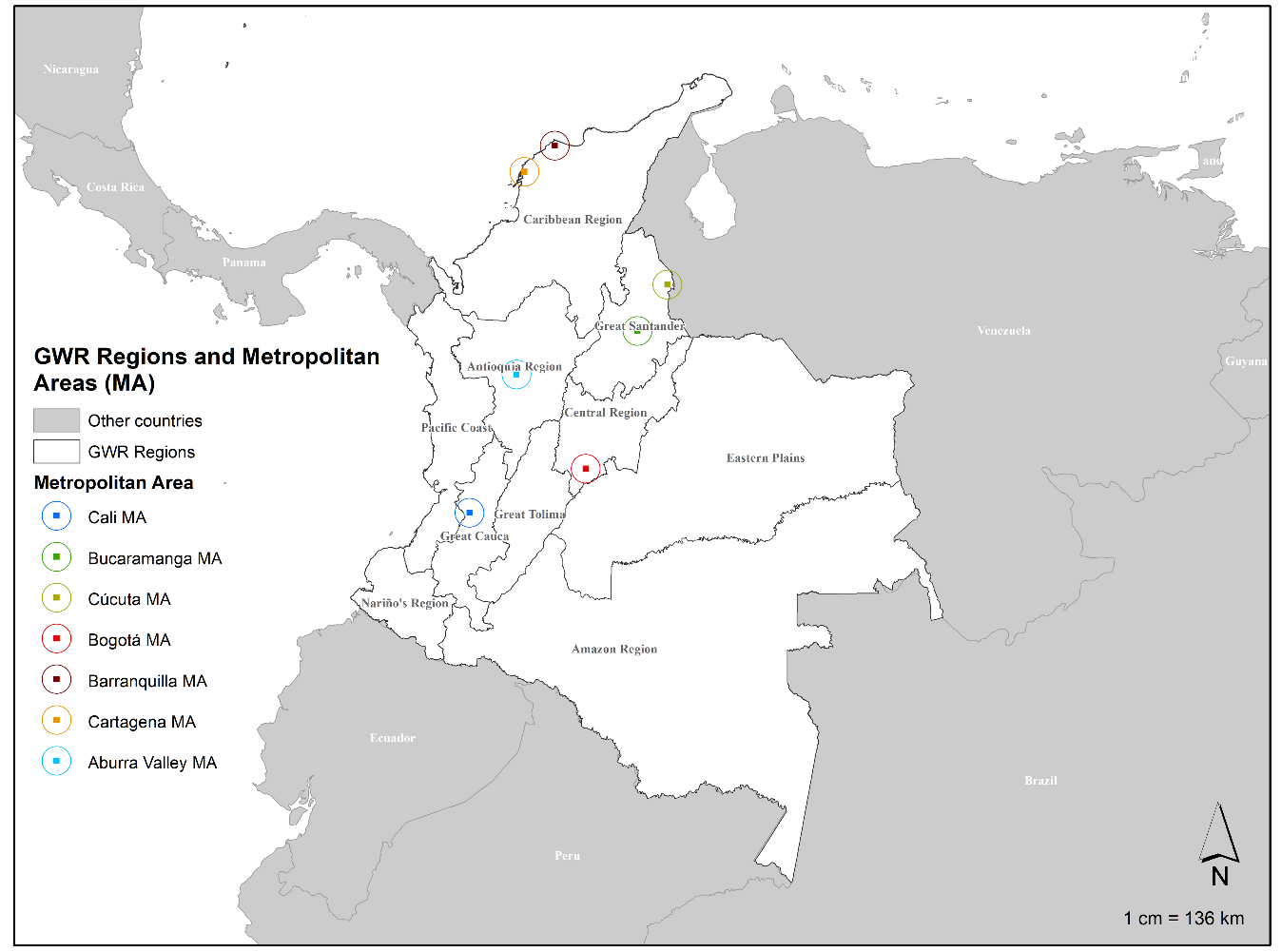


**Figure S-4**: Regions and metropolitan areas (MA) of Colombia used in the Geographically Weighted Regression analysis. This map was also build using the municipal open source shape file from The National Department of Statistics (DANE), which could be consulted and downloaded at the following link <https://geoportal.dane.gov.co/servicios/descarga-y-metadatos/descarga-mgn-marco-geoestadistico-nacional/>. International borders were obtained from ArcGis Hub open source data from the following link <https://hub.arcgis.com/datasets/a21fdb46d23e4ef896f31475217cbb08_1?geometry=-119.531%2C-89.983%2C119.531%2C-88.772>.

1. *Additional figures of regional GWR analysis*

| **Table S-1. GWR: One lag model over ApSE from 1991 to 1998 (T1)** | | | | | |
| --- | --- | --- | --- | --- | --- |
| Regions | Avg Local R2 | Intercept | Intercept's Std Error | FMF's Coefficient | FMF's *p-value* |
| Amazon Region | 0,308 | 0,484 | 0,065 | 3,678 | 0,000 |
| Nariño's Region | 0,350 | 0,652 | 0,063 | 3,033 | 0,000 |
| Great Tolima | 0,353 | 0,707 | 0,063 | 2,450 | 0,000 |
| Eastern Plains | 0,312 | 0,605 | 0,070 | 2,436 | 0,000 |
| Great Cauca | 0,362 | 0,695 | 0,061 | 2,323 | 0,000 |
| Central Region | 0,338 | 0,687 | 0,069 | 1,851 | 0,001 |
| Great Santander | 0,323 | 0,593 | 0,068 | 1,385 | 0,005 |
| Pacific Coast | 0,394 | 0,699 | 0,069 | 1,283 | 0,065 |
| Antioquia Region | 0,380 | 0,703 | 0,068 | 1,121 | 0,031 |
| Caribbean Coast | 0,392 | 0,555 | 0,065 | 0,824 | 0,058 |

**Table S-1.** Geographically Weighted Regression showing one lag model over ApSE from 1991 to 1998 (T2)

| **Table S-2. GWR: One lag model over ApSE from 1999 to 2006 (T2)** | | | | | |
| --- | --- | --- | --- | --- | --- |
| Regions | Avg Local R2 | Intercept | Intercept's Std Error | FMF's Coefficient | FMF's *p-value* |
| Amazon Region | 0,125 | 0,427 | 0,075 | 0,678 | 0,002 |
| Antioquia Region | 0,162 | 0,899 | 0,078 | 0,072 | 0,496 |
| Caribbean Coast | 0,227 | 0,902 | 0,075 | 0,092 | 0,303 |
| Central Region | 0,132 | 0,802 | 0,079 | 0,238 | 0,157 |
| Eastern Plains | 0,118 | 0,661 | 0,080 | 0,528 | 0,013 |
| Great Cauca | 0,101 | 0,562 | 0,071 | 0,242 | 0,054 |
| Great Santander | 0,168 | 0,853 | 0,078 | 0,134 | 0,291 |
| Great Tolima | 0,104 | 0,626 | 0,072 | 0,329 | 0,079 |
| Nariño's Region | 0,104 | 0,434 | 0,073 | 0,316 | 0,005 |
| Pacific Coast | 0,143 | 0,768 | 0,079 | 0,131 | 0,241 |

**Table S-2.** Geographically Weighted Regression showing one lag model over ApSE from 1999 to 2006 (T2)

| **Table S-3. GWR: One lag model over ApSE from 2006 to 2013 (T3)** | | | | | |
| --- | --- | --- | --- | --- | --- |
| Regions | Avg Local R2 | Intercept | Intercept's Std Error | FMF's Coefficient | FMF's *p-value* |
| Amazon Region | 0,235 | 0,665 | 0,085 | 0,358 | 0,000 |
| Antioquia Region | 0,322 | 1,303 | 0,087 | 0,058 | 0,274 |
| Caribbean Coast | 0,369 | 1,161 | 0,085 | 0,061 | 0,114 |
| Central Region | 0,271 | 1,181 | 0,088 | 0,125 | 0,010 |
| Eastern Plains | 0,239 | 1,004 | 0,089 | 0,219 | 0,001 |
| Great Cauca | 0,248 | 0,969 | 0,079 | 0,197 | 0,000 |
| Great Santander | 0,313 | 1,223 | 0,087 | 0,084 | 0,038 |
| Great Tolima | 0,245 | 1,000 | 0,081 | 0,213 | 0,002 |
| Nariño's Region | 0,244 | 0,830 | 0,082 | 0,255 | 0,000 |
| Pacific Coast | 0,308 | 1,198 | 0,088 | 0,091 | 0,240 |

**Table S-3.** Geographically Weighted Regression showing one lag model over ApSE from 2006 to 2013 (T3)


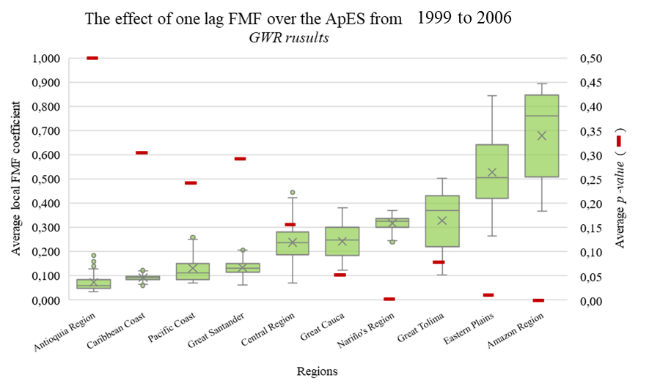


**Figure S-5.** GWR (Geographically Weighted Regression) detected coefficients of one lag FMF (Forced Migration Flow) over the ApES (Anthropogenic Print Spatial Expansion) from 1999 to 2006.

1. *Additional statistical tests*

To test for the robustness of our results, we first ran a Pearson correlation analysis between selected and additional demographic variables. We did this due to the concern that other possible demographic characteristics could bias the identified effect of forced migration over the urban expansion process in Colombia. In this sense, we took census data and official projections (2005 and 2018) and used them to calculate the average population growth rate (PGR). We found that PGR is uncorrelated with the percentage of urban population (correlation < 0.1) or with the demographic bonus (correlation < 0.03) and its quadratic form (correlation < 0.05).

We then used several specifications to test for both possible model dependence of our results and the potential bias of the FMF coefficients as a result of assuming the effect of the natural PGR of the municipalities within the "demographic bonus" variable. To do so, we ran the following models: two OLS with robust errors, one adding PGR and as an additional variable, and other without PGR; The other two models, were fixed estimations effects at the department-level with the same logic (see Table S-4). The rest variables, where the same as the one we used in estimated models showed in the main text.

As one can see in the results in Table S-4, the effect of migrations flows due to violence, seems to have a robust and significant (p < 0.01) positive effect on urban expansion (AsPE) across all study periods. Additionally, this effect does not substantially vary when accounting for population growth rate (PGR). Another observation is that when controlling for unobserved departmental characteristics, the effect of forced migration over urban expansion tends to decrease, but remains statically significant. Accordingly, our results and conclusions about the relationship between urban expansion (ApSE) and forced migration are consistent and robust, and thus offer an interesting path for further research. The effect of PGR over the Colombian urban expansion also seems very sensitive to the particular characteristics of each study period and changes in the model's structure. We also found that the effect of percentage of urban population and distance to the department's capital seems to have both stable and significant effects in the face of time and model variations.

**Table S-4. Additional models integrating new demographic variables and different model specifications.**

| **Robustness checks** | | | | | | | | | | | | | | | |
| --- | --- | --- | --- | --- | --- | --- | --- | --- | --- | --- | --- | --- | --- | --- | --- |
|  | ApSE from 1991 to 1998 | | | | | ApSE from 1999 to 2006 | | | | | ApSE from 2006 to 2013 | | | | |
| Variables | | OLS simple | OLS with PGR | Department FE | FE with PGR | | OLS simple | OLS with PGR | Department FE | FE with PGR | | OLS simple | OLS with PGR | Department FE | FE with PGR |
|  | |  |  |  |  | |  |  |  |  | |  |  |  |  |
| One lag FMF | | 1.540*** | 1.524*** | 0.711** | 0.705** | | 0.278*** | 0.251*** | 0.214*** | 0.215*** | | 0.160*** | 0.152*** | 0.075*** | 0.088*** |
|  |  | (0.362) | (0.357) | (0.356) | (0.353) | | (0.093) | (0.097) | (0.073) | (0.073) | | (0.034) | (0.033) | (0.027) | (0.026) |
| Demographic bonus | | -1.708*** | -1.752*** | -3.002*** | -2.969*** | | 0.549 | 0.288 | -0.877** | -0.878** | | -1.110** | -1.227*** | -2.105*** | -2.090*** |
|  |  | (0.380) | (0.368) | (0.350) | (0.346) | | (0.357) | (0.340) | (0.361) | (0.361) | | (0.472) | (0.451) | (0.378) | (0.373) |
| Demographic bonus squared | | 0.826*** | 0.853*** | 1.609*** | 1.600*** | | -0.363* | -0.194 | 0.520** | 0.521** | | 0.427 | 0.502* | 1.135*** | 1.128*** |
|  |  | (0.219) | (0.213) | (0.218) | (0.216) | | (0.213) | (0.204) | (0.221) | (0.222) | | (0.276) | (0.263) | (0.232) | (0.229) |
| Percentage of Urban Population | | 0.250*** | 0.251*** | 0.201*** | 0.188*** | | 0.155*** | 0.167*** | 0.110*** | 0.109*** | | 0.132*** | 0.137*** | 0.185*** | 0.170*** |
|  |  | (0.032) | (0.032) | (0.029) | (0.029) | | (0.031) | (0.031) | (0.032) | (0.032) | | (0.034) | (0.034) | (0.032) | (0.032) |
| ln(Distance to Departmental capital) | | -0.034*** | -0.033*** | -0.030*** | -0.032*** | | -0.060*** | -0.057*** | -0.046*** | -0.046*** | | -0.069*** | -0.068*** | -0.060*** | -0.060*** |
|  |  | (0.010) | (0.010) | (0.006) | (0.006) | | (0.011) | (0.010) | (0.007) | (0.007) | | (0.013) | (0.013) | (0.008) | (0.008) |
| Percentage of municipal area deforested | | 0.015 | 0.022 | 0.308* | 0.223 | | -0.088 | -0.013 | 0.110 | 0.104 | | -0.038 | -0.014 | 0.221 | 0.130 |
|  |  | (0.157) | (0.157) | (0.170) | (0.169) | | (0.194) | (0.190) | (0.177) | (0.178) | | (0.187) | (0.187) | (0.184) | (0.182) |
| Population Growth Rate (PGR) | |  | -0.166 |  | 1.296*** | |  | -1.389*** |  | 0.104 | |  | -0.580** |  | 1.591*** |
|  |  |  | (0.216) |  | (0.280) | |  | (0.223) |  | (0.295) | |  | (0.293) |  | (0.302) |
| Constant | | 1.004*** | 1.020*** | 1.508*** | 1.492*** | | 0.405*** | 0.497*** | 0.919*** | 0.919*** | | 1.190*** | 1.231*** | 1.451*** | 1.443*** |
|  |  | (0.158) | (0.152) | (0.139) | (0.138) | | (0.148) | (0.138) | (0.145) | (0.145) | | (0.193) | (0.183) | (0.152) | (0.150) |
|  | |  |  |  |  | |  |  |  |  | |  |  |  |  |
| Observations | | 1,041 | 1,041 | 1,041 | 1,041 | | 1,096 | 1,096 | 1,096 | 1,096 | | 1,109 | 1,109 | 1,109 | 1,109 |
| R-squared | | 0.343 | 0.343 | 0.478 | 0.489 | | 0.147 | 0.170 | 0.350 | 0.350 | | 0.259 | 0.262 | 0.511 | 0.524 |
| AIC | | -704.6 | -703.2 | -944.9 | -964.9 | | -374.8 | -403.9 | -672.9 | -671.1 | | -99.30 | -101.5 | -560.5 | -586.9 |
| BIC | | -670 | -663.6 | -910.2 | -925.3 | | -339.8 | -363.9 | -637.9 | -631.1 | | -64.20 | -61.40 | -525.5 | -546.9 |
| Log-likelihood | | 359.3 | 359.6 | 479.4 | 490.5 | | 194.4 | 209.9 | 343.5 | 343.5 | | 56.63 | 58.73 | 287.3 | 301.5 |
| Robust standard errors in parentheses | | |  |  |  | |  |  |  |  | |  |  |  |  |
| *** p<0.01, ** p<0.05, * p<0.1 | | |  |  |  | |  |  |  |  | |  |  |  |  |

**Table S-4.** Additional models integrating new demographic variables and different model specifications**.**

Although the RUV database is officially recognized nationally and intentionally (see International Displacement. Monitoring Center (IDMC) 2020, Colombia: Country information, accessed 15 June 2020, <https://www.internal-displacement.org/countries/colombia>.), it is also essential to contrast these data against other sources. However, to our knowledge, the RUV database is the only one with the following characteristics, it: disaggregates at the municipal level, differentiated between expulsion and reception of people, distributes migration flows in annual intervals since the 1980s, and focuses exclusively on forced migration derived from the armed conflict, and not on other migration phenomena. That said, our intent in not incorporating other databases into our analyses is not to ignore the debates that have taken place regarding the real magnitudes of forced displacement

Accordingly, other additional databases were reviewed and considered. The first was the ELCA (longitudinal survey of Colombia by the Universidad de los Andes) survey that periodically monitors various individuals and families in the country and that contains a special section for families who were victims of forced displacement. However, although the ELCA allows for characterizing these families' living conditions, it is not an instrument that allows accounting for the country's annual net forced migration in such a way that it enables estimating the models used in this analysis. Another two databases were also consulted including the IDMC (The Internal Displacement Monitoring Center) and the OCHA (UN Office for the Coordination of Humanitarian Affairs). However, both databases base their estimation of forced migration on the RUV database and are not desegregated at the required municipal level. Accordingly, we decided to calculate the population growth due to internal migration (GIM), based on the census data from 2018 (which could be consulted at DANE, population estimates 1985–2005 and population projections 2005–2020 total municipal by area, from the following link <https://www.dane.gov.co/index.php/estadisticas-por-tema/demografia-y-poblacion/proyecciones-de-poblacion> and Municipal population estimates by area 2005 - 2017 based on the CNPV 2018, from the following link <https://www.dane.gov.co/files/censo2018/proyecciones-de-poblacion/anexo-Estimaciones_de_Poblacion_2005-2017-Mpiosxarea.xlsx>), taking two different questions from data:

1. Age: A categorical variable within five-year groups.
2. Birthplace: Enables one to identify if a person is from the municipality in which they were surveyed, from another municipality (not specifying which one) or from another country.

To calculate the GIM, we made two assumptions. The first assumption is that the probability of migrating is a constant overall population at any given time, and the second one is that the probability of migrating is constant in time over each age rank. As we are taking census data to estimate past migration flows, we have both a restriction of not counting migrants who have died before the census and not counting migrants who migrate again before the poll.

The assumption behind equation S-10 is that a migrant of a given age, initially has the same probability of migrating at any given previous year. However, we observed that the probability of being a migrant is different for each age group. The probability of migrating between 0 and 4 years of age is different from the probability of migrating between 40 and 44 years old). Therefore, we assume that migrating at a certain period depends on the probability of migrating at a certain age that a person had at that particular time using Eq S-11.

$Pr\left( Migrating \right)_{it}=Pr(Migraiting|{Age}_{it})$ (Equation S-10)

${GIM}_{tm}=\frac{Pr({Migating}_{t}|{Age}_{a})*\sum_{a}^{A} Migrant Population}{Total pulation_{tm}}$ (Equation S-11)

Therefore, to estimate the GIM (percentage of population growth due to internal migration) for each period, we group migrant population by age ranks ("a"), multiplying it by the probability of migrating on any period ("t"; given the age that each group had on each period). Finally, we add these results at the municipal level and divide them by the total population of each municipality ("m") at that given time (Equation S-11). Total population at any given time was obtained from population projections both from the national census of 2005 and 1985. In doing so we used census data from all available Colombian censuses. Table S-5, shows the estimated total internal migration for each study period and the descriptive statistics of the GIM. We note that these estimations are about internal migration independent of its source (forced or not forced).

| Study Period | Total Estimate Migration | GIM | | | |
| --- | --- | --- | --- | --- | --- |
|  |  | Mean | Std. Dev. | Min | Max |
| T1 | 2,691,652 | 5.0% | 3.8% | 0.0% | 36.2% |
| T2 | 4,181,104 | 9.1% | 6.5% | 0.0% | 93.3% |
| T3 | 5,497,139 | 12.9% | 6.8% | 0.2% | 60.0% |

**Table S-5. General statistics of the estimation of total internal migration and population growth due to internal migration (GIM) from census data.**

After estimating internal migration from census data, we ran the same models (fixed effect at departmental level), but integrating this new variable (GIM) as shown in table S-6. We observed that the effect of FMF over ApSE stays positive and significant for every study period. These additional models show that there is a statistically significant relationship between forced migration and the expansion of urban prints across Colombia during our study periods. Our models also capture most (56%) of the variation of ApSE, presenting evidence of a plausible causal path that could be studied in the future.

|  | T1 | T2 | T3 |
| --- | --- | --- | --- |
| Variables | 1991 to 1998 | 1999 to 2006 | 2006 to 2013 |
|  |  |  |  |
| One lag FMF | 0.627* | 0.213*** | 0.054** |
|  | (0.334) | (0.073) | (0.025) |
| GIM | 1.990*** | 0.109 | 1.138*** |
|  | (0.169) | (0.102) | (0.098) |
| Demographic bonus | -2.154*** | -0.820** | -1.528*** |
|  | (0.336) | (0.365) | (0.360) |
| Demographic bonus squared | 1.145*** | 0.488** | 0.830*** |
|  | (0.208) | (0.223) | (0.220) |
| Percentage of Urban Population | 0.114*** | 0.108*** | 0.180*** |
|  | (0.028) | (0.032) | (0.030) |
| ln(Distance to Departmental capital) | -0.024*** | -0.046*** | -0.057*** |
|  | (0.006) | (0.007) | (0.007) |
| Percentage of municipal area deforested | -0.119 | 0.065 | -0.278 |
|  | (0.164) | (0.182) | (0.179) |
| Constant | 1.068*** | 0.887*** | 1.061*** |
|  | (0.136) | (0.148) | (0.147) |
|  |  |  |  |
| Observations | 1,041 | 1,096 | 1,109 |
| R-squared | 0.542 | 0.350 | 0.566 |
| AIC | -1078 | -672.1 | -689.3 |
| BIC | -1038 | -632.1 | -649.2 |
| Log-likelihood | 547 | 344.1 | 352.7 |
| Standard errors in parentheses | | | |
| *** p<0.01, ** p<0.05, * p<0.1 | | | |

**Table S-6. Additional fixed effect models (by department) integrating estimated Growth by Internal Migration (GIM).**
